# Fluoxetine Potentiates Phagocytosis and Autophagy in Microglia

**DOI:** 10.3389/fphar.2021.770610

**Published:** 2021-11-24

**Authors:** Sung Hee Park, Young-Sun Lee, Hyun-Jeong Yang, Gyun Jee Song

**Affiliations:** ^1^ Department of Medical Science, Catholic Kwandong University College of Medicine, Gangneung, Korea; ^2^ The Convergence Institute of Healthcare and Medical Science, Catholic Kwandong University, International St. Mary’s Hospital, Incheon, Korea; ^3^ Department of Integrative Biosciences, University of Brain Education, Cheonan, Korea

**Keywords:** fluoxetine, microglia, phagocytosis, autophagy, neuroinflammation

## Abstract

Fluoxetine is a classic antidepressant drug, and its immunomodulatory effects have recently been reported in many disease models. In addition, it has strong antineuroinflammatory effects in stroke and neurodegenerative animal models. However, the effect of fluoxetine on microglia phagocytosis and its molecular mechanisms have not yet been studied. In this study, we investigated whether fluoxetine has a regulatory effect on microglial function. Microglia cell lines and primary mouse microglia were treated with fluoxetine, and the production of inflammatory cytokines and neurotrophic factors and the phagocytosis of amyloid β were measured. Fluoxetine significantly attenuated the production of lipopolysaccharide-induced proinflammatory cytokines and oxidative stress in microglia. Fluoxetine also significantly potentiated microglia phagocytosis and autophagy. In addition, autophagy flux inhibitors attenuated fluoxetine-induced phagocytosis. In conclusion, fluoxetine induces autophagy and potentiates phagocytosis in microglia, which can be a novel molecular mechanism of the neuroinflammatory and neuroprotective effects of fluoxetine.

## Introduction

Microglia are innate immune cells of the central nervous system (CNS), and resting microglia in the mature brain are known to play a role in brain homeostasis (Lenz and Nelson, 2018). Microglia is very sensitive to the changes in the microenvironment, and they can be activated in response to infection or injury-induced molecules. Activated microglia cause morphological changes and an increase in surface receptors and release several types of substances (Song and Suk, 2017; Zhao et al., 2017). Among them, there are neurotrophic factors that affect the survival of neurons and have neuroprotective effects (Song et al., 2016a; Song et al., 2016b; Ramirez et al., 2017). Activated microglia also produce many proinflammatory and neurotoxic factors (Jha et al., 2018; Song et al., 2019; Gupta et al., 2020). These proinflammatory cytokines and molecules produced by these inflammatory activated microglia cause neuroinflammation, which can lead to neurodegenerative diseases (Ramirez et al., 2017; Hansen et al., 2018). For example, the treatment of neurons with tumor necrosis factor (TNF-α), interleukin 1β (IL-1β), and nitric oxide (NO) was reported to cause the loss of synapses and neuronal death, suggesting that the inflammatory activation of microglia plays an important role in neurodegenerative disease pathogenesis. Conversely, there are neuroprotective types of microglia (Kwon and Koh, 2020) that show anti-inflammatory and regenerative functions. Often known as M2 microglia, these cells can relieve inflammation through the secretion of anti-inflammatory molecules, mainly inhibit the production of proinflammatory cytokines, and release neurotrophic factors such as brain-derived neurotrophic factor (BDNF) and insulin-like growth factor 1 (Chen and Trapp, 2016). In addition, M2 microglia have been reported to clear tissue debris and misfolded proteins to maintain CNS homeostasis as CNS phagocytes.

Autophagy is one of the key intracellular functions that maintain cellular homeostasis by removing misfolded proteins and damaged organelles. As the aggregation of misfolded proteins and mitochondrial dysfunction are the main etiology of most neurodegenerative diseases, autophagy dysfunction is recently considered to play an important role in many neurodegenerative diseases (Plaza-Zabala et al., 2017; Corti et al., 2020). Therefore, it has been proposed that autophagy regulation in neurons may have a therapeutic effect in neurodegenerative diseases. More recently, the dysregulation of autophagy in microglia has been reported to contribute to neurodegenerative disease pathogenesis (Menzies et al., 2017; Cho et al., 2020). For example, autophagy disorders in microglia can affect immune functions such as phagocytosis or neuroinflammation (Plaza-Zabala et al., 2017). However, whether autophagy activation regulates phagocytosis in microglia remains uninvestigated.

Fluoxetine is selective serotonin reuptake inhibitors (SSRIs), which increases the extracellular level of serotonin by limiting its reuptake into the presynaptic cell, and is mainly used as a treatment for depressive syndrome. Serotonin is also known to affect immune regulation via different 5-hydroxytryptamine (5-HT) receptors (5-HTRs) expressed in cells of the innate and adaptive immune system (Herr et al., 2017). Serotonin inhibited the production of TNF-α in Lipopolysaccharide (LPS)-stimulated mononuclear cells, with effects inhibited by 5-HT2 receptor blockade (de las Casas-Engel et al., 2013; Herr et al., 2017). Fluoxetine can act as an agonist to the 5-HT2B (Peng et al., 2014), and 5-HT2B was detected at the mRNA level in microglia collected from adult mice (Kettenmann et al., 2011; Turkin et al., 2021). Therefore, SSRIs such as fluoxetine have anti-inflammatory effects in microglia through 5-HT2B; however, the mechanisms related to other microglial functions, such as phagocytosis, remain unknown.

Recently, fluoxetine has been reported to reduce neuroinflammation *in vitro* and *in vivo* animal models (Liu et al., 2011; Zhang et al., 2012; Liu et al., 2018). Fluoxetine reduces neuroinflammation by decreasing proinflammatory cytokine production (TNF-α, IL-1β, IL-6, etc.), phosphorylation of inducible NO synthase (iNOS), p38 mitogen-activated protein kinase, and nuclear factor κB (NF-κB) activity. It also reduces hippocampal neuronal damage caused by cerebral hemorrhage and has neuroprotective effects such as neurogenesis and the induction of cell proliferation (Khodanovich et al., 2018). Furthermore, fluoxetine has an autophagy induction capacity in other cell types. A few studies have shown that, when administered in disease models, fluoxetine causes autophagic activation (Sun et al., 2018).

In this study, we investigated whether fluoxetine has anti-inflammatory effects and induces autophagy and phagocytosis in microglia.

## Materials and Methods

### Cell Culture

BV-2 cells, an immortalized mouse microglial cell line, were maintained in Dulbecco modified eagle medium (DMEM; Gibco, Grand Island, NY, USA) with 5% fetal bovine serum (FBS; Gibco) and 100 U/mL penicillin/streptomycin (Gibco). HAPI cells, an immortalized rat microglial cell line, were cultured in DMEM with 10% FBS and 100 U/mL penicillin/streptomycin at 37°C in a 5% CO_2_ incubator.

Primary mixed glial cells (MGCs) were prepared from neonatal C57BL/6 mice on postnatal days 1–3, as previously described (Gupta et al., 2020), with minor modifications. All experiments were conducted in accordance with the institutional animal care committee of the Catholic Kwandong University (no. CKU 2020-012). The cell suspensions obtained from brain tissue dissections were cultured with DMEM supplemented with 10% FBS and 100 U/mL penicillin/streptomycin for 3 weeks, with medium changes every 3 days.

### Microglia Isolation From Adult Mouse Brain

Primary mouse microglial cultures were performed using a mouse adult brain dissociation and microglia isolation kit (MACS; Mitenyi Biotec, Bergisch Gladbach, Germany). Briefly, the brains of wild-type C57Bl6/J mice (3 months old) were removed immediately after euthanasia and digested with digestion buffer (Miltenyi Biotec, 130-107-677) at 37°C for 40 min. After the removal of myelin debris, the cell suspension was filtered through a 70-μm cell strainer. Cell pellets were washed with phosphate-buffered saline (PBS) with 0.5% bovine serum albumin (BSA), followed by CD11b-positive selection (Miltenyi Biotec, 130-093-636) using a mini-MACS (Miltenyi Biotec, 130-042-201) column. CD11b-positive microglia were seeded on poly-l-lysine–coated coverslips and cultured in DMEM with 10% FBS.

### Measurement of Nitric Oxide and Cell Viability Assay

The cells were plated on 96-well plates (4 × 10^4^/well) in full serum DMEM media. The cells were treated with or without concentrations of fluoxetine (Sigma–Aldrich, St. Louis, MO, USA), in the absence or presence of LPS (100 ng/mL; Sigma–Aldrich), in serum-free media for 24 h. NO production was quantified by adding a Griess solution, and the absorbance was measured at 540 nm with a microplate reader. Standard curves were prepared based on the reference values of a serially diluted sodium nitrite solution. Cell viability was determined using the 3-[4, 5-dimethylthiazol-2-yl]-2,5-diphenyl tetrazolium bromide (MTT; Sigma–Aldrich) assay. Absorbance was measured at 570 nm.

### Reverse Transcription–Polymerase Chain Reaction

Total RNA was extracted from the treated cells using the TRIZOL reagent (Invitrogen, Carlsbad, CA, USA), and cDNA was synthesized using Moloney murine leukemia virus reverse transcriptase (Promega) and oligo(dT) primers. Reverse transcription–polymerase chain reaction (RT-PCR) was performed with specific primer sets, as shown in Table 1, using a T100 Thermal Cycler (Bio-Rad Laboratories, Richmond, CA, USA). PCR products were detected under ultraviolet light following ethidium bromide (Sigma–Aldrich) staining.

**TABLE 1:** Primers used for RT-PCR.

Target genes	Accession number	Forward primer (5′–3′)	Reverse primer (5′–3′)	Temp (°C)	Cycles
TNF-α	NM_013693.2	CAT​CTT​CTC​AAA​ATT​CGA​GTG​ACA​A	ACT​TGG​GCA​GAT​TGA​CCT​CAG	60	24
IL-1β	NM_008361.4	GCAACTGTTCCTGAACTC	CTCGGAGCCTGTAGTGCA	60	29
IL-6	NM_031168.2	AGT​TGC​TTC​TTG​GGA​CTG​A	TCC​ACG​ATT​TCC​CAG​AGA​AC	57	27
Arg-1	NM_007482	CGC​CTT​TCT​CAA​AAG​GAC​AG	CCA​GCT​CTT​CAT​TGG​CTT​TC	60	29
BDNF	NM_007540.4	CGC​AAA​CAT​GTC​TAT​GAG​GGT​TC	TAG​TAA​GGG​CCC​GAA​CAT​ACG​AT	60	30
GAPDH	NM_008084	ACC​ACA​GTC​CAT​GCC​ATC​AC	TCC​ACC​ACC​CTG​TTG​CTG​TA	60	24

### Western Blot Assay

MGCs were treated with the following chemicals: fluoxetine (7.5 µM), 3-MA (50 µM; Sigma–Aldrich), chloroquine (CQ; 50 µM; Sigma–Aldrich), and LPS (100 ng/mL; Sigma–Aldrich) for 3 h. The cells were washed with cold PBS after various treatments and lysed with a RIPA lysis buffer (50 mM Tris-HCl, 150 mM NaCl, 0.1% sodium dodecyl sulfate (SDS), and 1% NP-40). Equal amounts of protein were separated on a 12% SDS–polyacrylamide gel and transferred to polyvinylidene difluoride membranes (Bio-Rad Laboratories). The blots were blocked with 5% skim milk for 1 h at room temperature and incubated with the following primary antibodies: rabbit antimicrotubule-associated protein light chain 3 (LC3; 1:1,000 dilution; MBL, Woburn, MA, USA) or mouse anti–β-actin (1:5,000 dilution; Sigma–Aldrich, St. Louis, MO, USA) overnight at 4°C. After washing, the membranes were incubated for 1 h at room temperature with horseradish peroxidase–conjugated secondary antibodies (1:2,000) in 5% skim milk. The blots were developed using an enhanced chemiluminescence detection kit (SuperSignal™ West Femto; Thermo Fisher, Franklin, MA, USA).

### Phagocytosis Assay

The phagocytosis assay was performed with fluorescent zymosan bioparticles from *Saccharomyces cerevisiae* (pH-sensitive pHrodoTM Red dye conjugates; Life Technologies, Carlsbad, CA, USA) or oligomerized FITC–amyloid-β (M-2585; BACHEM, Bubendorf, Switzerland). Primary MGCs were seeded at a density of 2 × 10^4^ cells/well in 96-well plates and cultured for 48 h. The cells were treated with LPS (1 μg/mL) and/or fluoxetine (5 µM) and/or bafilomycin (Baf; 10 nM; Sigma–Aldrich) for 24 h in serum-free DMEM. The cells were treated with zymosan red or FITC–amyloid-β in serum-free media for 2–3 h. In some experiments, we conducted time-lapse imaging using a fluorescence microscope (DP80; Olympus, Japan). Blindly labeled images were analyzed to quantify phagocytosis.

### Immunofluorescence Assay

The cells were cultured on coverslips and stained for LC3 or Iba-1 expression. Briefly, the cells were fixed in 4% paraformaldehyde (Sigma–Aldrich) for 20 min and blocked with 1% BSA (Sigma–Aldrich) and 4% normal donkey serum with 0.1% Triton X-100 (Sigma–Aldrich) in PBS (PBST) for 1 h at room temperature. The cells were incubated with primary rabbit anti-LC3 (1:500, MBL, Nagoya, Japan) or rabbit anti–Iba-1 (1:200, Wako, Neuss, Germany) in 1% BSA in PBST at 4°C overnight and then incubated with Cy3-conjugated secondary antibody (1:500 dilutions, Jackson ImmunoResearch, West Grove, PA, USA) for 1 h. The coverslips were mounted using a VECTASHIELD^®^ antifade mounting solution with DAPI (Vector Laboratories, Burlingame, CA, USA). Blindly labeled images were analyzed to quantify LC3 punta.

### Statistical Analysis

Data are presented as mean ± standard error of the mean (SEM) from at least three independent experiments. Statistical significance was analyzed using unpaired Student *t* test to compare two groups and one-way analysis of variance (ANOVA; Tukey *post hoc* multiple-comparisons tests) for multiple groups. *p* < 0.05 was considered statistically significant.

## Results

### Fluoxetine Inhibits the Production of LPS-Induced Proinflammatory Molecules in Microglia

**FIGURE 1:**
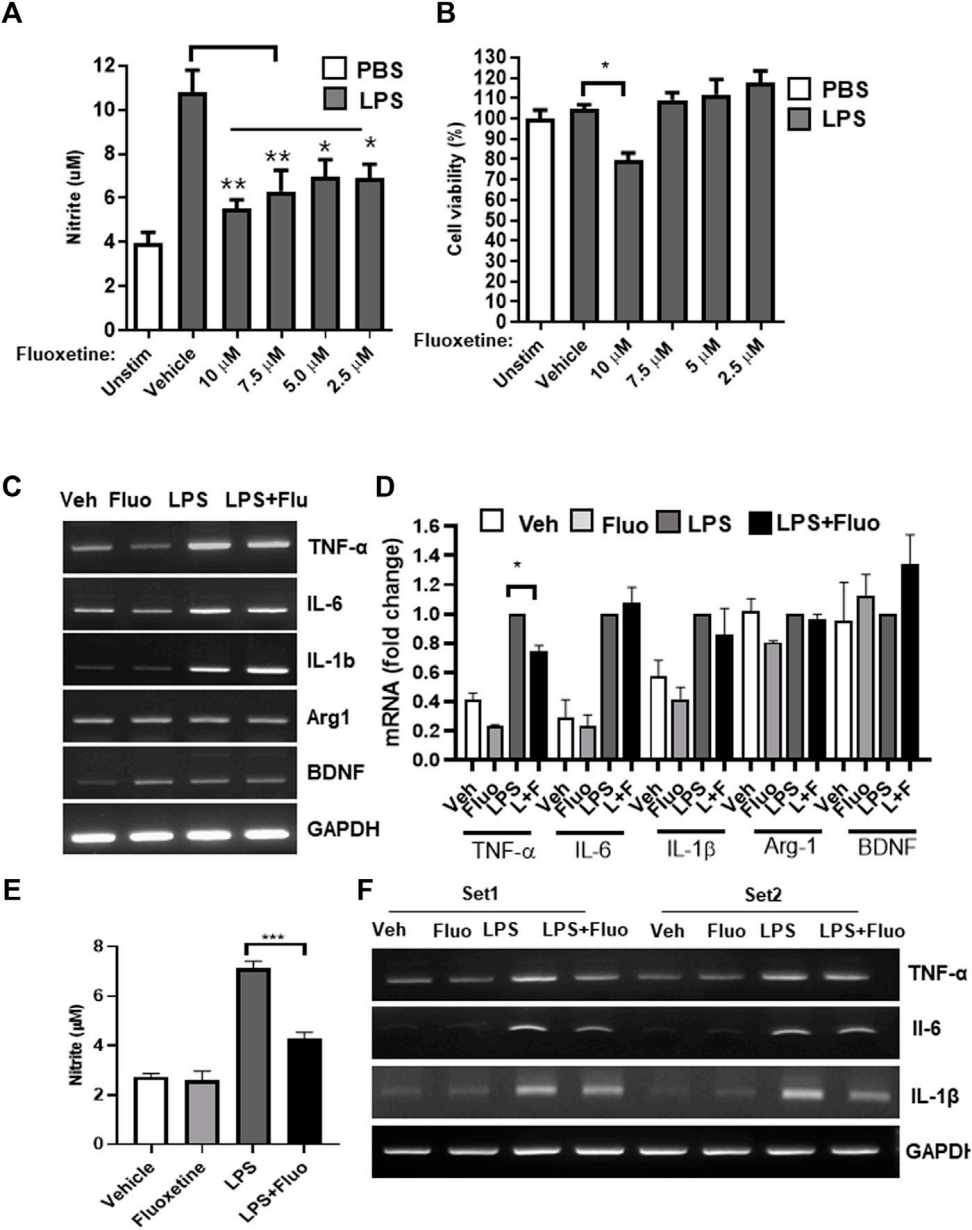
Fluoxetine reduces inflammation in microglia. BV-2 cells were treated with the indicated concentrations of fluoxetine with or without LPS (100 ng/mL). After 24 h, NO production was measured using the Griess assay **(A)**, and cell viability was measured using the MTT assay **(B)**. **(C**, **D)** BV-2 cells were treated with 7.5 μM fluoxetine (fluo) with or without LPS. After 24 h, the mRNA expression levels of TNF-α, IL-6, IL-1β, Arg1, and BDNF were determined using RT-PCR. Fold changes were calculated as the ratio of the expression level in the LPS-only treated group. **(E)** A rat microglia cell line, HAPI cells, was also used to confirm the anti-inflammatory effect of fluoxetine. The cells were treated with 7.5 μM fluoxetine with or without LPS. NO production was measured after 24 h of incubation. **(F)** HAPI cells were treated with 7.5 μM fluoxetine for 6 h with or without LPS, and mRNA levels of inflammatory cytokines were measured using RT-PCR. Data are presented as the mean ± SEM. *p < 0.05, **p < 0.01, ***p < 0.001 compared with the LPS-only treated group in an one-way ANOVA with Tukey *post hoc* multiple-comparisons test. Veh, Vehicle. One-way ANOVA: **(A)** *F*(5,18) = 9.387, *p* = 0.0002; **(B)** *F*(5,18) = 7.624, *p* = 0.0005; **(D)** *F*(3,8) = 134.8, *p* < 0.0001; **(E)** *F*(3,12) = 57.97, *p* < 0.0001.

The anti-inflammatory effect of fluoxetine was examined in the microglial cell line BV-2. When BV-2 cells were treated with LPS, NO increased, and when fluoxetine was treated with LPS, NO production was significantly inhibited in a concentration-dependent manner. A slight toxicity was observed at high concentrations (10 μM). Therefore, other experiments were conducted at nontoxic concentrations (7.5 and 5 μM) of fluoxetine with NO inhibition effect (Figures 1A, B). Next, we have investigated whether fluoxetine modulates the expression of the neurotoxic or neuroprotective genes in microglia. Fluoxetine treatment significantly reduced TNF-α mRNA production, an inflammatory cytokine in BV-2 (Figures 1C, D), but no significant differences in the expression of Arg1, an M2 microglia marker, and BDNF, a neurotrophic factor, were observed. This anti-inflammatory effect was also confirmed in HAPI cells, another microglia cell line (Figures 1E, F).

### Fluoxetine Promotes Phagocytosis in Microglia

**FIGURE 2:**
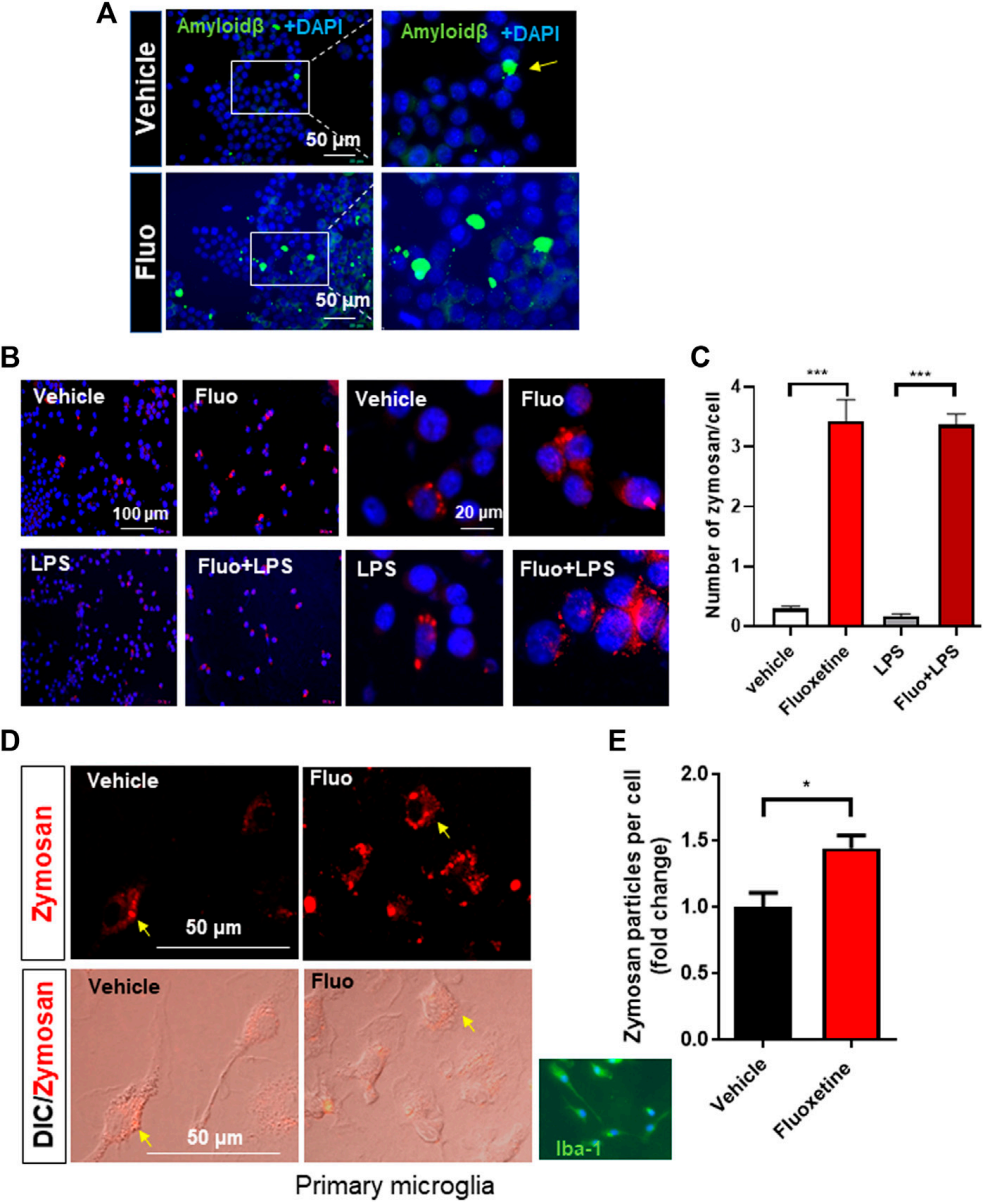
Fluoxetine enhances phagocytosis in microglia. **(A)** BV-2 cells were seeded on coverslips in a 24-well plate and incubated overnight. The cells were then treated with fluoxetine (Fluo; 7.5 μM) for 16 h, followed by incubation with oligomerized amyloid β-GFP (100 µM in DMEM) for 3 h. The phagocytosed amyloid β-GFP was imaged under the fluorescence microscope. **(B)** BV-2 cells were treated with fluoxetine (7.5 μM) with or without LPS (100 ng/mL) for 16 h. The cells were then incubated with opsonized zymosan red particles for another 3 h. **(C)** The phagocytosed zymosan particles were counted and expressed as the number of zymosan particles per cell. More than 325 cells per group (total of >1,300 cells) were analyzed for quantification. **(D)** Primary microglia isolated from adult mice (Iba-1 microglial marker stained in green) was treated with fluoxetine (5 μM) for 24 h. The cells were then incubated with opsonized zymosan red particles for another 3 h. **(E)** The phagocytosed zymosan particles were counted and expressed as the fold change of the number of zymosan particles per cell. A total of 250 cells (117 and 133 cells per group) were analyzed for quantification. Data are presented as the mean ± SEM. *p < 0.05, ***p < 0.001 compared with fluoxetine-treated cells in ANOVA with Tukey multiple-comparisons test or Student *t* test. One-way ANOVA: **(C)** *F*(3,40) = 83.38, *p* < 0.0001.

One of the important functions of microglia is the clearance of pathogens and aggregated proteins (Galloway et al., 2019). Therefore, in this study, we analyzed whether fluoxetine increases microglial phagocytosis. First, the phagocytosis of fluorescently labeled amyloid-β was observed in BV-2. Cells pretreated with fluoxetine were incubated with oligomerized amyloid β_1-42_-GFP, and GFP engulfed by microglia was observed under a fluorescence microscope. As shown in Figure 2A, the phagocytosis of amyloid β_1-42_-GFP increased with fluoxetine pretreatment. To digest phagocytosed particles, phagosomes fuse to lysosomes to form phagolysosomes. To determine whether fluoxetine increases phagolysosome formation, cells were treated with pHrodo-Red–labeled zymosan. When zymosan enters the phagolysosome, an organelle with a low pH, it turns into a strongly red fluorescent particle. As a result of counting and quantifying zymosan particles per cell, it was confirmed that phagocytosis significantly increased with fluoxetine in BV-2 in the presence or absence of LPS (Figures 2B, C). Next, primary microglia were used to confirm fluoxetine-induced phagocytosis. After isolating microglia from the adult mouse brain using a CD11b antibody, adult microglia were pretreated with fluoxetine for 24 h and then treated with zymosan particles for 3 h to obtain a fluorescence image. As shown in Figures 2D, E, red phagocytosed zymosan particles rapidly increased after entering the cell by fluoxetine.

### Fluoxetine Increases Autophagy in Microglia

**FIGURE 3:**
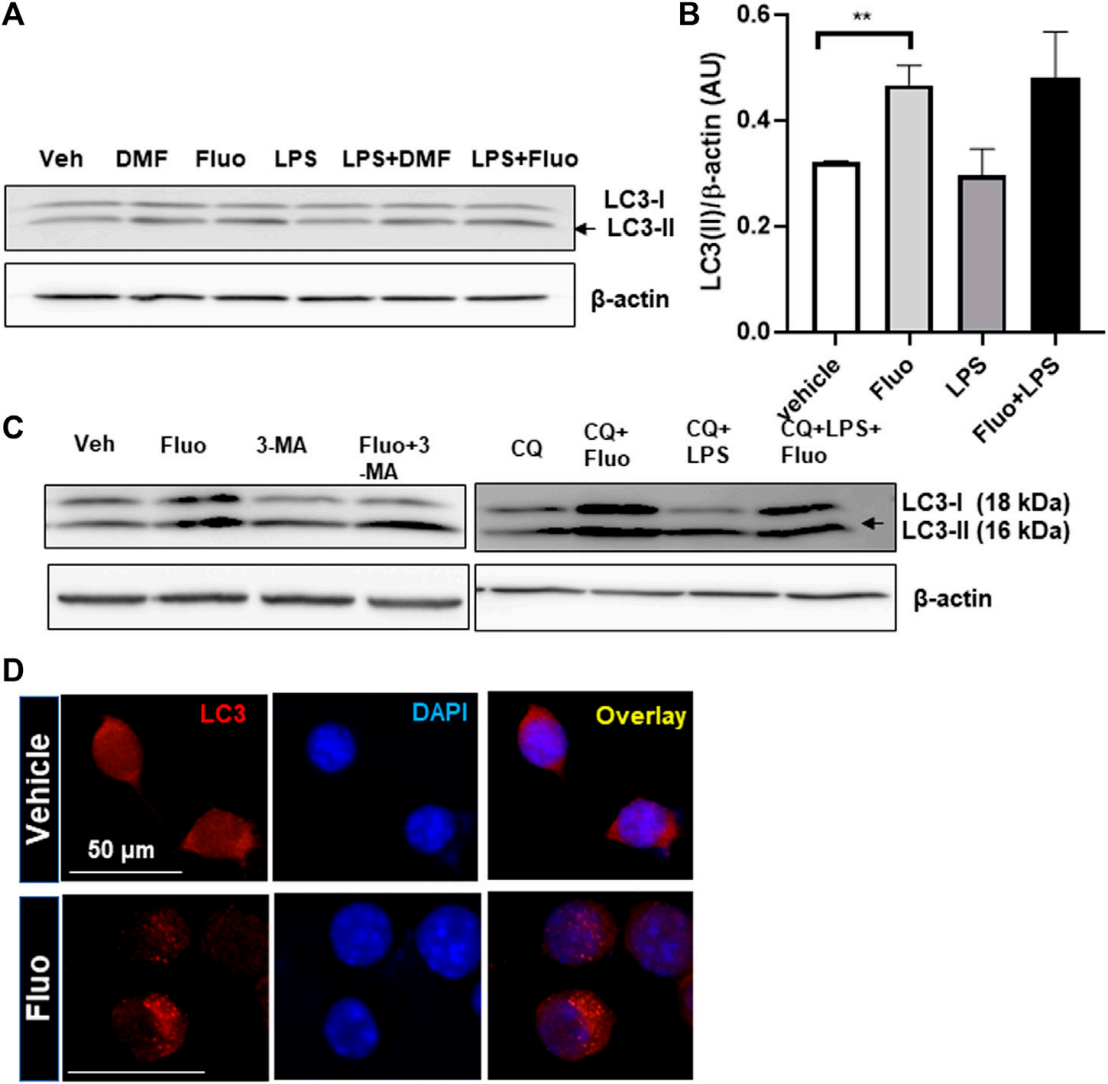
Fluoxetine enhances autophagy in microglia. **(A)** HAPI cells were treated with DM*F*(4 μM), fluoxetine (7.5 μM), and/or LPS (100 ng/mL), as indicated, for 3 h. Cell lysates were immunoblotted for LC3-I and LC3-II. β-Actin was used as a loading control. **(B)** LC3-II quantification. Band intensity was quantified using ImageJ, and LC3-II band intensities were normalized based on β-actin band intensities. One-way ANOVA [*F*(3,12) = 4.720, *p* = 0.0213, n = 3–5/group]. The differences were not significant between groups after Tukey multiple-comparisons tests. Data are presented as means ± SEM. **p < 0.01 compared with fluoxetine-treated cells in an unpaired *t* test. **(C)** Autophagy induction in primary MGCs isolated from the mouse brain with fluoxetine treatment. MGCs were treated with the following chemicals: fluoxetine (7.5 μM), 3-MA (50 μM), CQ (50 μM), and LPS (100 ng/mL) for 3 h. Cell lysates were immunoblotted for LC3-I and LC3-II. **(D)** Autophagosome formation labeled by LC3 in microglia. BV-2 cells were treated with fluoxetine (7.5 μ) for 3 h and immunostained for LC3 in red. Images were obtained with a 40× fluorescent microscope.

Autophagy is known to be involved in the regulation of microglial function (Lee et al., 2021). Therefore, we examined whether fluoxetine induces autophagy in microglia. First, microglia were treated with fluoxetine, and the levels of autophagosome protein LC3-I and LC3-II were measured using Western blot. We found that LC3-II accumulation (LC3-II/loading control β-actin) was increased by fluoxetine treatment in both cells with or without LPS treatment (Figures 3A, B). DMF, an autophagy inducer, was used as a positive control (Lee et al., 2021). To determine whether fluoxetine increases the autophagy flux, we perturbed it by treatment with its inhibitor CQ. The fluoxetine (7.5 µM)–induced LC3-II increase was potentiated under CQ treatment (Figure 3C). Autophagosome formation in microglia was visualized by immunofluorescence, which revealed that the LC3 puncta per cell were dramatically increased in fluoxetine-treated BV-2 cells (Figure 3D). Our data suggest that fluoxetine contributes to autophagy induction and flux.

### Autophagy Inhibitor Decreases Fluoxetine-Induced Phagocytosis

**FIGURE 4:**
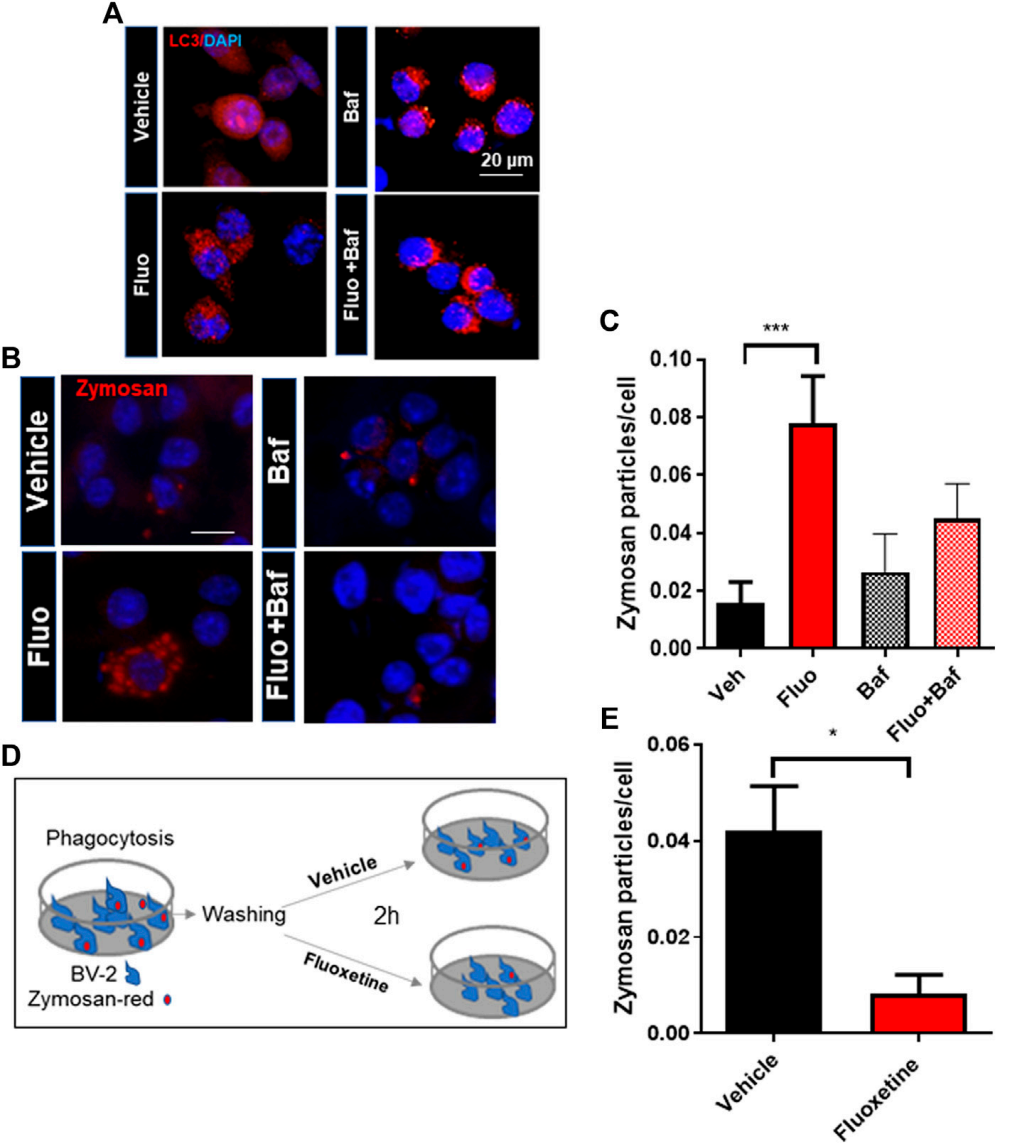
The autophagy inhibitor decreases fluoxetine-induced phagocytosis. **(A)** Autophagosome formation visualized by LC3 immunostaining in BV-2 cells with fluoxetine (7.5 μM) and/or Baf (10 nM) for 3 h. **(B)** Fluoxetine-induced phagocytosis blocked by an autophagy flux inhibitor, Baf (10 nM). BV-2 cells were pretreated with fluoxetine (7.5 μM) for 3 h and then treated with zymosan red for another 3 h with and without Baf. The cells were imaged under a fluorescence microscope. **(C)** The phagocytosed zymosan particles were counted and expressed as the number of zymosan particles per cell. A total of 1,850 cells (520, 560, 370, and 400 per group) were analyzed for quantification. **(D**, **E)** Fluoxetine potentiates the clearance of phagocytosed materials. BV-2 cells were incubated with zymosan red for 3 h and then washed three times. The cells were further incubated with either vehicle or fluoxetine for 2 h to measure the clearance of phagocytosed zymosan particles. The fixed cells were imaged using fluorescence microscopy. Data are presented as means ± SEM. *p < 0.05, ***p < 0.001 compared with fluoxetine-treated cells in ANOVA [*F*(3,32) = 4.408, *p* = 0.0105] with Tukey multiple-comparisons test **(C)** or Student *t* test **(E)**.

To determine whether autophagy is required for the pro-phagocytic effects of fluoxetine, we perturbed the phagosome flux by treatment with Baf. Baf treatment increased LC3 puncta formation by inhibiting the autophagy flux (Figure 4A). The phagocytosis assay showed that Baf significantly inhibits fluoxetine-induced phagocytosis (Figures 4B, C). To test whether fluoxetine-induced phagocytosis potentiates the clearance of pathogens or phagocytosed materials, the cells were treated with zymosan fluorescence particles first and then with or without fluoxetine for another 3 h to induce clearance (Figure 4D). As shown in Figure 4E, fluoxetine rapidly removed the phagocytic zymosan particles. These results support the finding that an enhanced autophagic activity contributes to phagocytosis and the clearance of phagocytosed materials. Taken together, these results demonstrate that fluoxetine could contribute to autophagy activation to engulf and digest pathogens or misfolded proteins, which could exert neuroprotective effects.

## Discussion

Fluoxetine, an SSRI, is commonly prescribed to treat depression. Recent studies have suggested that fluoxetine affords strong neuroprotective ability in many neurological disease models. However, the molecular mechanisms underlying fluoxetine-mediated neuroprotection have not yet been elucidated. In the present study, fluoxetine was shown to have antineuroinflammatory and prophagocytic effects. In particular, the results of fluoxetine’s effects and mechanisms of action are reported in microglia, which are responsible for maintaining brain immunity and homeostasis. Fluoxetine not only inhibits the proinflammatory activity of microglia but also increases phagocytosis and autophagy. The effects of inhibiting brain inflammation and reducing the accumulation of misfolded protein aggregates are expected to be helpful in the treatment of neurodegenerative diseases such as AD and PD.

Fluoxetine was developed as an antidepressant, but its anti-inflammatory and brain-protective effects have also been reported. Previous studies on the effects of fluoxetine on microglia are consistent with our findings. According to an article reported by Lui et al. (2011) in *Neuropharmacology*, fluoxetine significantly inhibited LPS-stimulated proinflammatory cytokines and neurotoxic mediators in BV-2 cells, as in our study (Figure 1). In addition, fluoxetine exerts a neuroprotective effect against microglia-mediated neurotoxicity in neuron and glia cocultures (Zhang et al., 2012). Fluoxetine has neuroprotective effects by reducing the production of proinflammatory factors, including NO, TNF-α, iNOS, and IL-1β, in early brain injury after subarachnoid hemorrhage (Liu et al., 2018). As a potential mechanism of the antineuroinflammatory effect of fluoxetine, fluoxetine-mediated NF-κB regulation has been proposed. Moreover, fluoxetine has been reported to display beneficial effects in models of neurological disorders, which may not be related to antineuroinflammatory effects (Gassen et al., 2015).

Recent studies have shown that fluoxetine is involved in the induction of autophagy, as summarized in a review article (Cho et al., 2020). Autophagy declines with age, and its deficit has been observed in the brain of many neurodegenerative diseases. Treatment with autophagy-inducing drugs led to a robust recovery rate and decline in clinical symptoms in rodent models of neurodegenerative diseases (Rusmini et al., 2019). Our recently published article also showed autophagy induction by DMF, reduced neuroinflammation by the inhibition of the M1 phenotype, and an increase in the M2 phenotype of microglia (Lee et al., 2021). Fluoxetine treatment exhibits autophagy induction in lymphoma (Cloonan and Williams, 2011) and adipose-derived stem cells (Sun et al., 2015). It significantly up-regulated autophagy-related genes and LC3 protein expression (Sun et al., 2015). Gassen et al. (2015) reported that fluoxetine activates autophagic pathways in an FKBP51-dependent manner. More recently, fluoxetine was found to activate autophagy in a rat subarachnoid hemorrhage brain injury model (Liu et al., 2018). In addition, fluoxetine reversed depressive behavior and up-regulated BDNF and autophagy-associated proteins (LC3-II) in normal mice. However, microglia-specific autophagy-deficient mice showed higher inflammatory levels and reduced BDNF expression (Tan et al., 2018). In their study, fluoxetine increased the expression of autophagy-related proteins ATG5, LC3-II, and BDNF, suggesting that fluoxetine-induced autophagy in the brain. Furthermore, Cx3Cr1Cre/ATG5loxp/loxp mice demonstrated a significantly lowered fluoxetine-induced BDNF overexpression. Interestingly, mouse brains with a microglia-specific autophagy deficiency showed the hyperactivation of microglia, as examined by Iba-1 staining in the hippocampal area of the brain. However, this study did not provide a mechanistic explanation of how fluoxetine mediates antidepressant effects through the autophagic pathway.

In this study, we showed the autophagy induction effect of fluoxetine in microglia. Serotonin and citalopram, another type of SSRI, are also known to have autophagy induction capacity in other cell types (Zschocke et al., 2011; Niture et al., 2018). In addition, SSRIs, such as escitalopram, fluoxetine, sertraline, paroxetine, and venlafaxine, are known to have an anti-inflammatory role (Dionisie et al., 2021). However, whether other members of SSRIs have anti-inflammatory effects and the ability to induce autophagy and phagocytosis in microglia, or whether these are fluoxetine-specific effects, is unclear. Further research on these topics is warranted.

Phagocytosis of dead cell debris and pathogens has been reported to be essential for the maintenance of brain homeostasis. Its dysfunction in microglia has been reported in patients with neurodegenerative diseases (Galloway et al., 2019). Recently, Berglund et al. (2020) reported that the deletion of Atg7 in microglia caused persistent neuroinflammation by driving microglial dysfunction in debris uptake and degradation. The reduced clearance capacity of microglia is most likely a consequence of the internalization of scavenger receptors due to impaired ATG7-dependent lysosomal degradation. The accumulation of intracellular proteins, including myelin and amyloid β, is associated with neurogenerative pathologies. ATG7-deficient microglia showed similar phenotypes to those of aged microglia. Therefore, molecules promoting the induction of autophagy might have a therapeutic potential. In this study, fluoxetine increased the canonical autophagosome formation examined by LC3 puncta and increases in LC3-II accumulation after treatment with CQ, an autophagy flux inhibitor.

The phagocytic capacity of microglia is essential for brain homeostasis maintenance. Especially, the dysfunction of microglia has been proposed as the common histology in aged brains and those with neurodegenerative diseases. Therefore, boosting the phagocytic capacity of microglia increases the clearance of neurotoxic proteins such as β-amyloid, α-synuclein, and TDP-43 (Boland et al., 2018). In our study, we found that fluoxetine has a prophagocytic function, a key function of microglia for maintaining brain homeostasis. We also showed that fluoxetine-induced phagocytosis is dependent on fluoxetine-mediated autolysophagosome formation in microglia.

Together, our findings demonstrate that fluoxetine has regulatory effects on autophagy and phagocytosis, which are necessary functions for microglia and CNS cellular homeostasis. We provide a novel mechanism for the neuroprotective and antineuroinflammatory effects of fluoxetine, which serves as a functional link between autophagy and phagocytosis capacity of microglia. We suggest that fluoxetine is an autophagic inducer in microglia and potentiates their phagocytic capacity. The initial step of autophagy is autophagosome formation, which fuses with lysosomal vesicles and mediates the delivery of cytoplasmic proteins to lysosomal vesicles to be degraded or recycled. Fluoxetine-induced autophagy increased LC3 puncta formation, as well as lysophagosome formation. An enhanced autophagic activity has been reported to promote Aβ clearance *in vitro* and in AD mice. Here, we find that fluoxetine treatment caused autophagic activation, as seen by increased LC3-II and LC3 punctate distribution and autophagosome accumulation. Furthermore, the fluoxetine-mediated increase in phagocytosis was blocked by the autophagy inhibitor Baf in microglia. Collectively, these data indicate that fluoxetine exerts an anti-inflammatory and neuroprotective effect in the brain through microglia, suggesting that modulating the autophagy–lysosomal pathway can be a promising therapy for the clearance of amyloid plaques in AD.

In conclusion, our study demonstrated that fluoxetine attenuated neuroinflammation and potentiated phagocytosis in microglia. A potential mechanism of fluoxetine-induced phagocytosis is, at least, partially involved in the activation of autophagy in microglia.

## Data Availability

The original contributions presented in the study are included in the article, further inquiries can be directed to the corresponding author.
